# Supplementary material for: phuEGO: A Network-Based Method to Reconstruct Active Signaling Pathways From Phosphoproteomics Datasets
Source: Mol Cell Proteomics. 2024 Apr 19;23(6):100771. doi: 10.1016/j.mcpro.2024.100771 (PMC11134849; doi:10.1016/j.mcpro.2024.100771)
Supplement: Supplemental Figures [file mmc3.pdf]

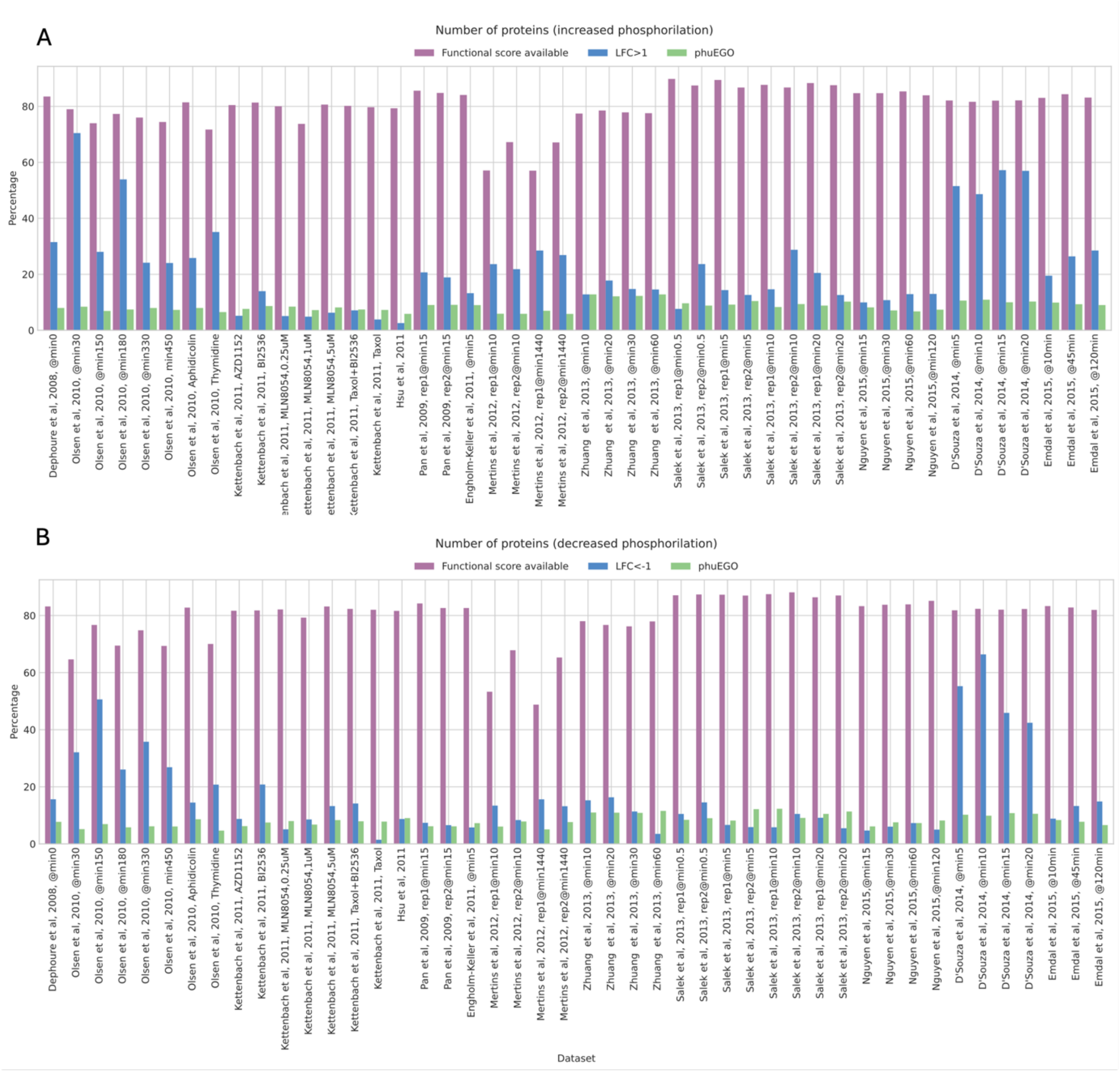

Supplementary Figure 1. Number of proteins: Phosphosites annotated with a functional score, phosphosites with LFC>1 and proteins in phuEGO networks for each dataset analysed in Figure 2 & Supplementary Figure 7. A. For upregulated phosphosites **B.** For downregulated phosphosites

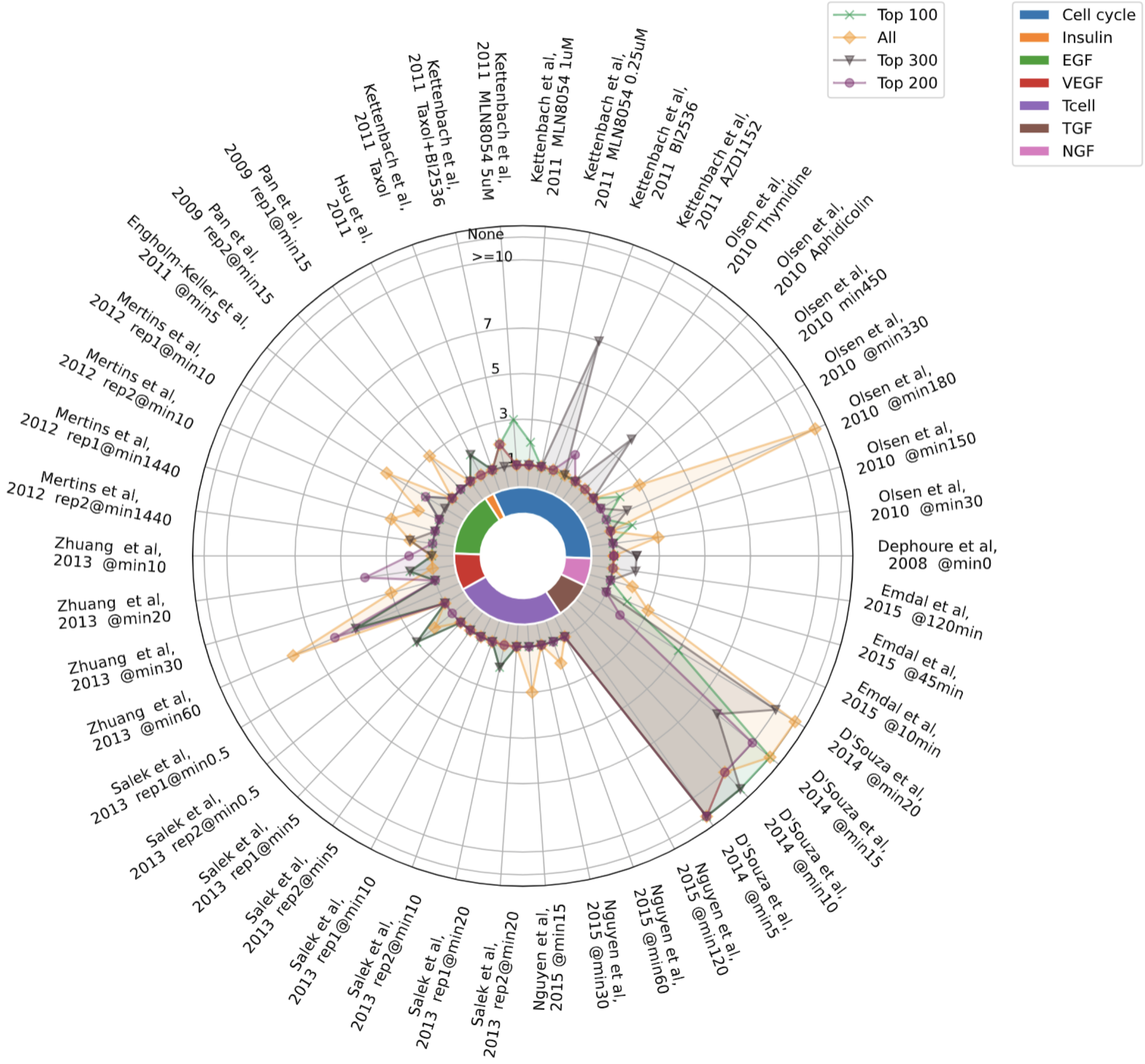

Supplementary Figure 2. Replication of Main Figure 2 using different numbers of input seed nodes.

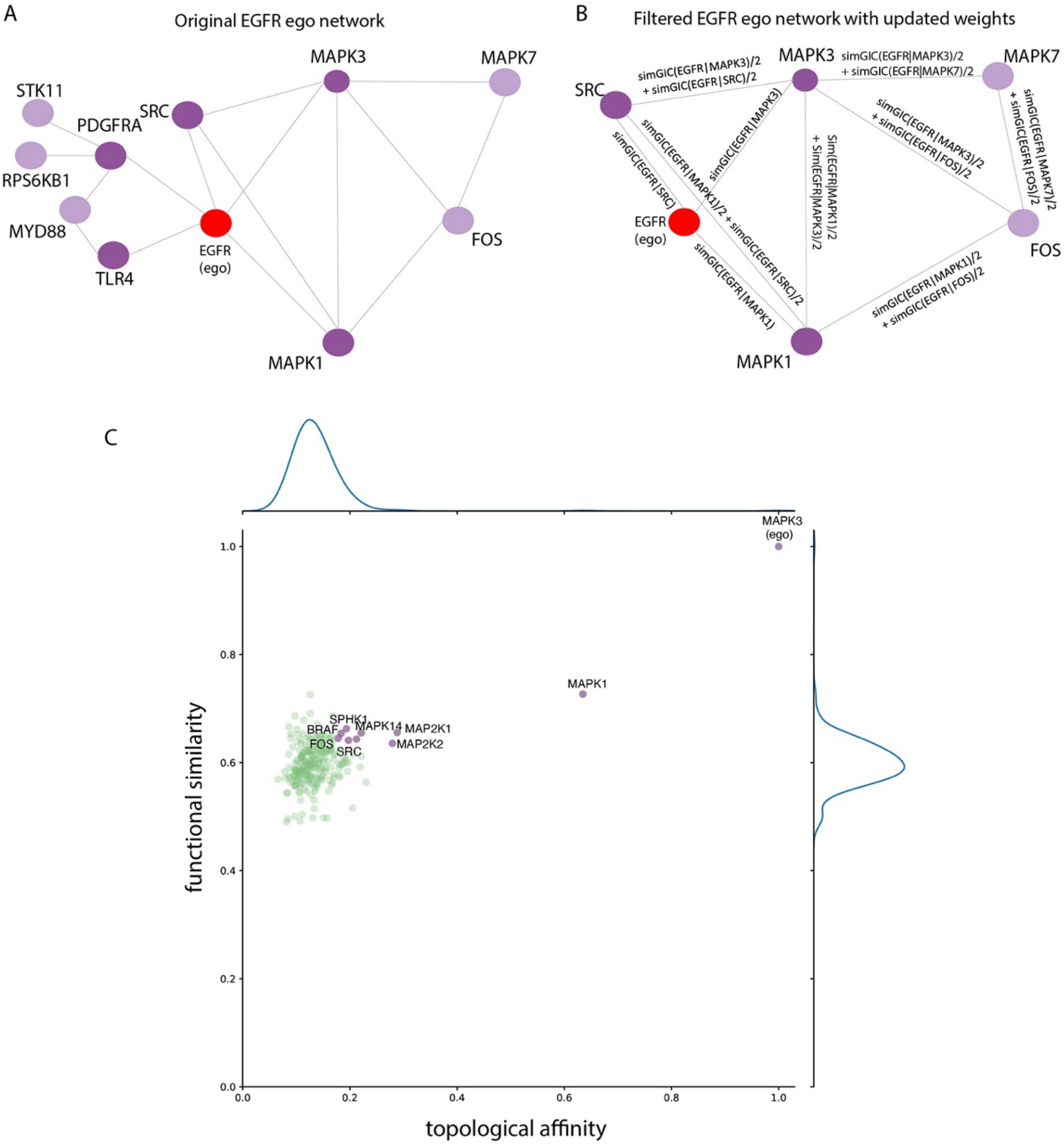

Supplementary Figure 3. Schematic of generation of EGO networks A. Example of using EGFR as a seed generation to generate the initial EGO network **B.** Example of filtering the network using the topological and semantic similarity. **C.** Example of using the Kernel Density Estimation to identify the most topologically and functionally similar nodes to the network.

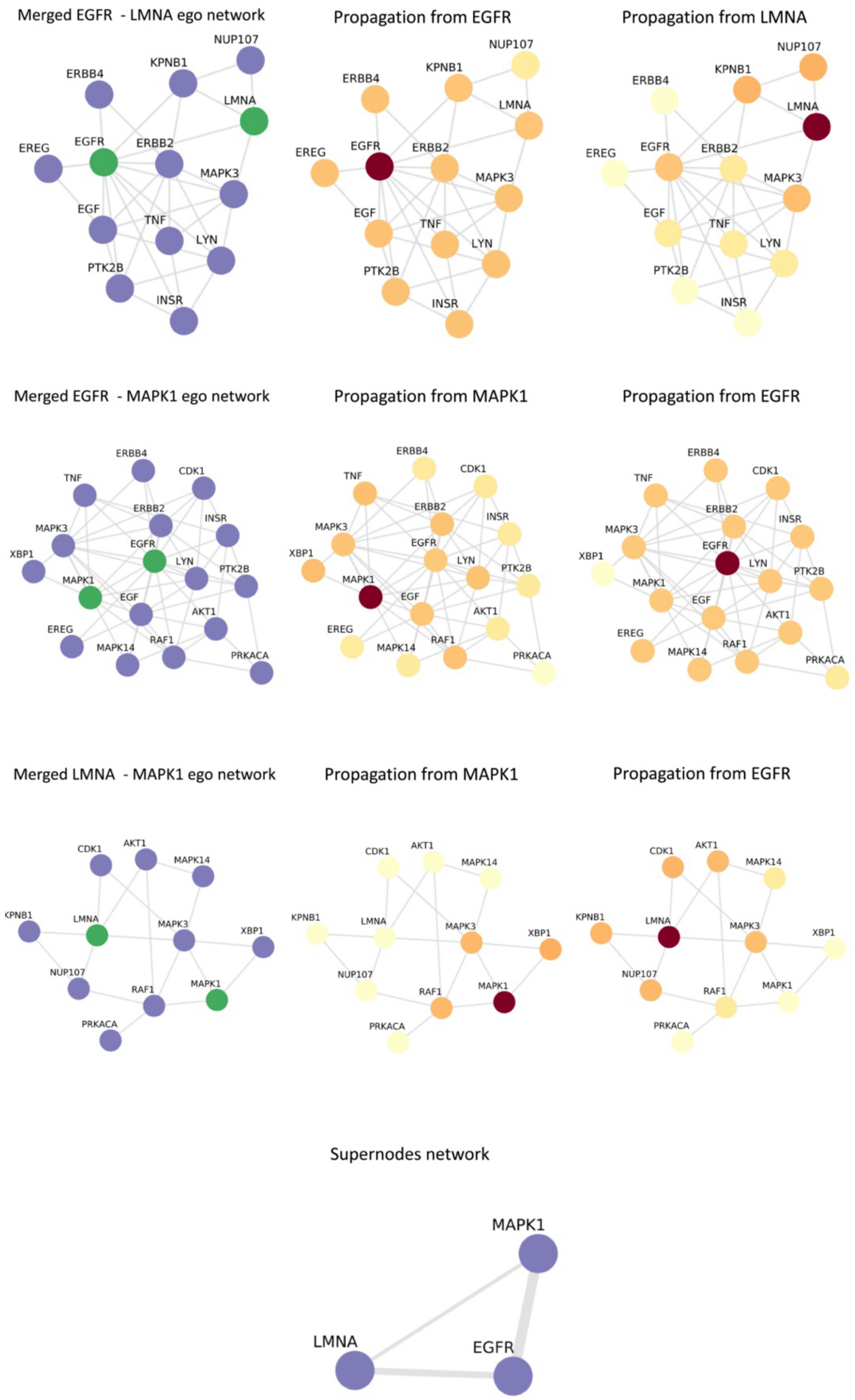

Supplementary Figure 4. Schematic of merging EGO networks into a supernode network

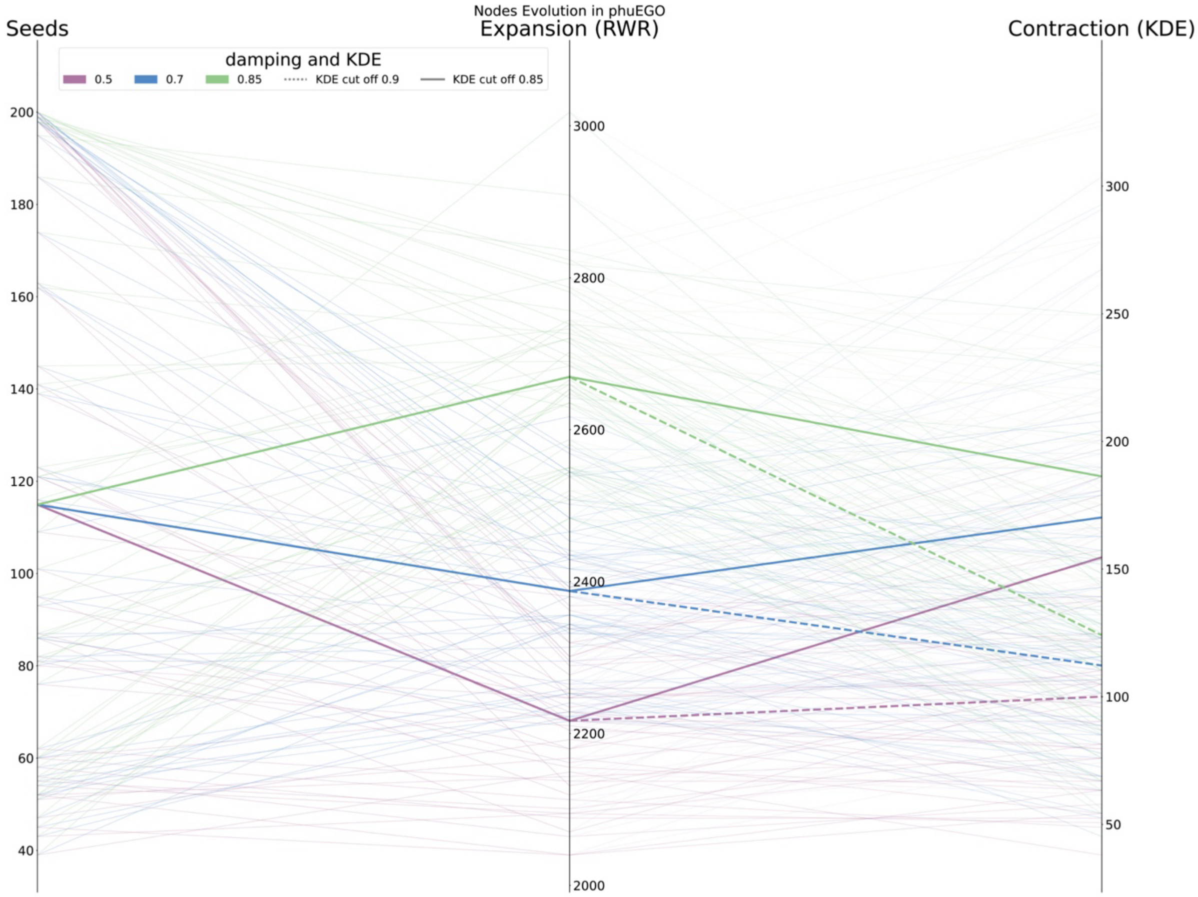

Supplementary Figure 5. Changes in network size at the different steps of phuEGO.

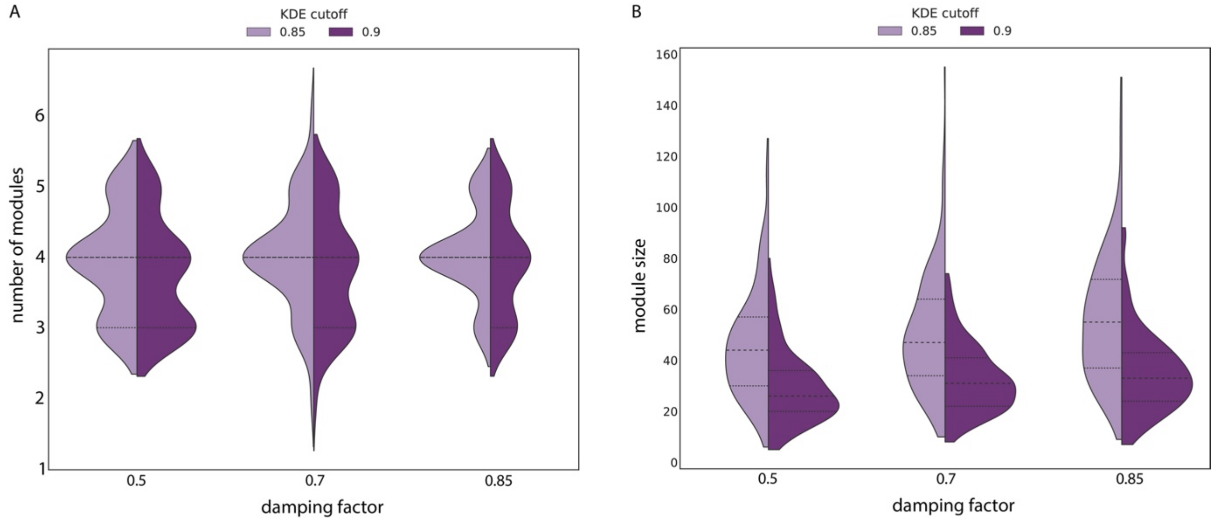

Supplementary Figure 6. Characteristics of phuEGO-extracted modules A. Distribution of number of modules in the datasets tested in this study **B.** Distribution of module sizes generated from the datasets tested in this study.

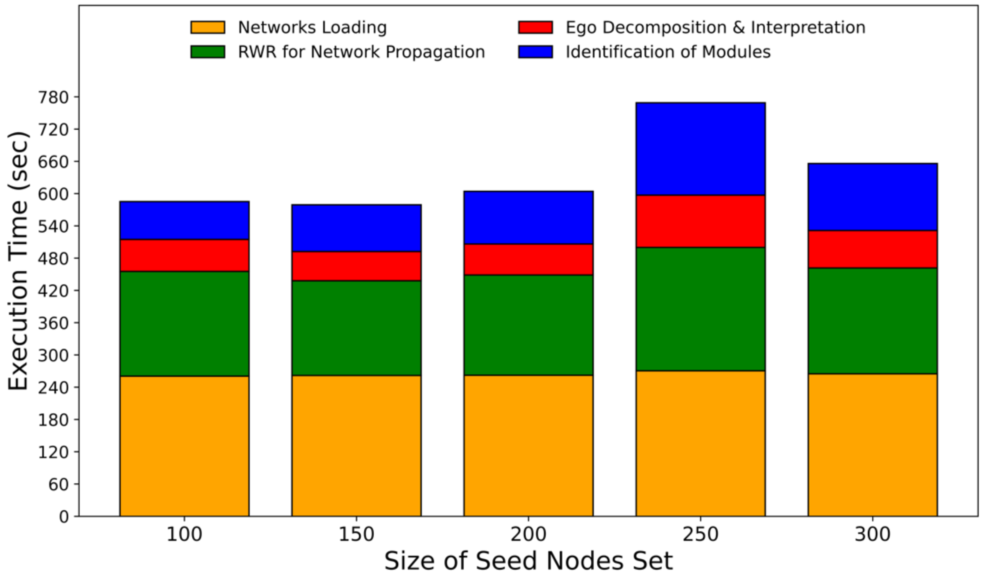

Supplementary Figure 7. Runtime of algorithm and distribution of time across the relevant tasks.

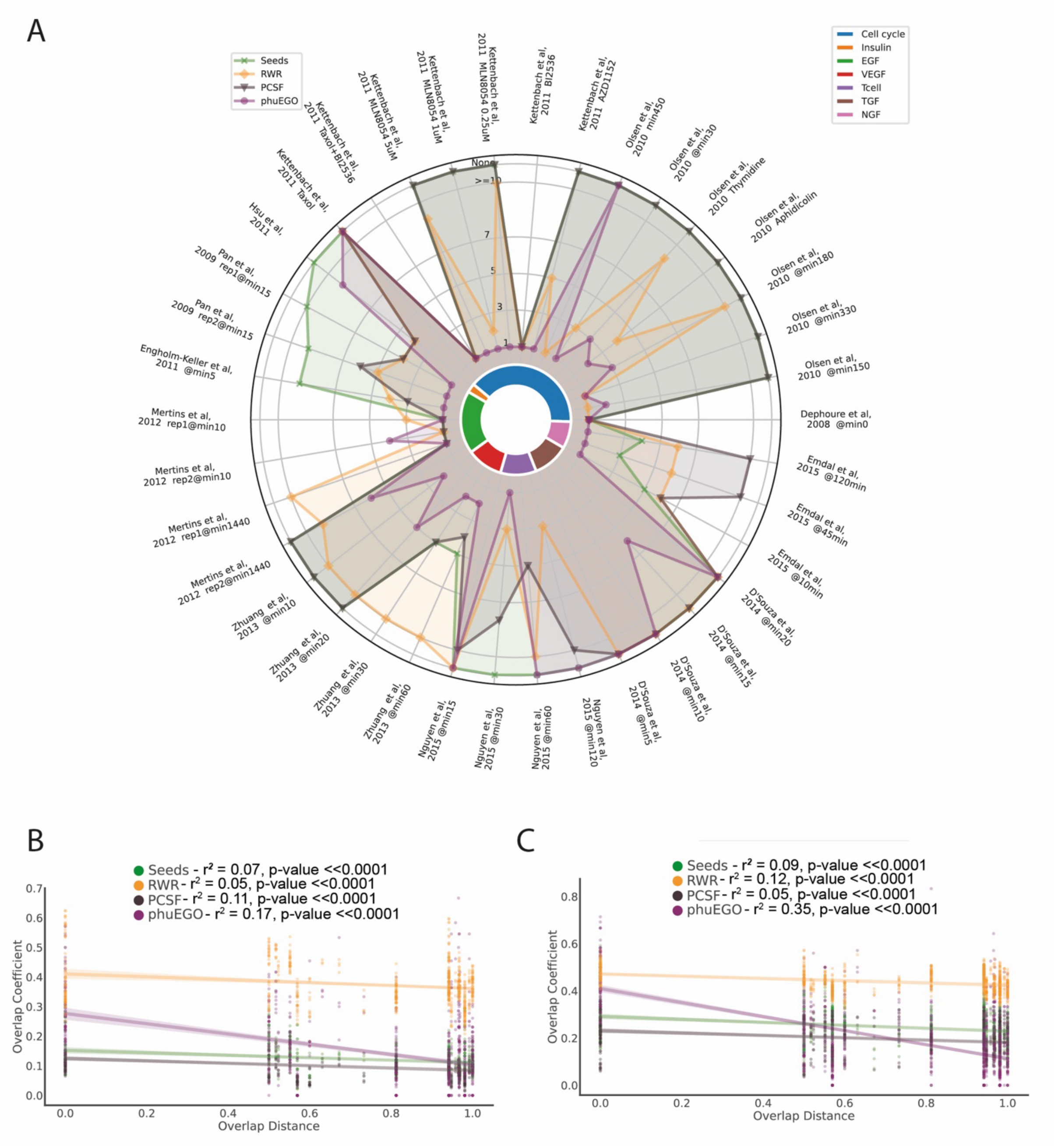

Supplementary Figure 8. Evaluation of phuEGO on phosphoproteomics datasets derived from the original publications A. Comparison of seeds, PCSF, RWR and phuEGO with respect to their ability to rank highly the expected dominant signal, as defined by pathway enrichment analysis. The centre of the circle indicates the relevant pathway ranked first and the perimeter indicates a failure to identify the pathway at any rank. **B & C.** Association of the overlap coefficient of the phuEGO active signature with the overlap distance of the expected pathways across all pairwise pathway comparisons in our benchmark test **B.** in the original datasets used in the Ochoa et al, 2019 study **C.** in the uniformly processed data in the Ochoa et al, 2019 study

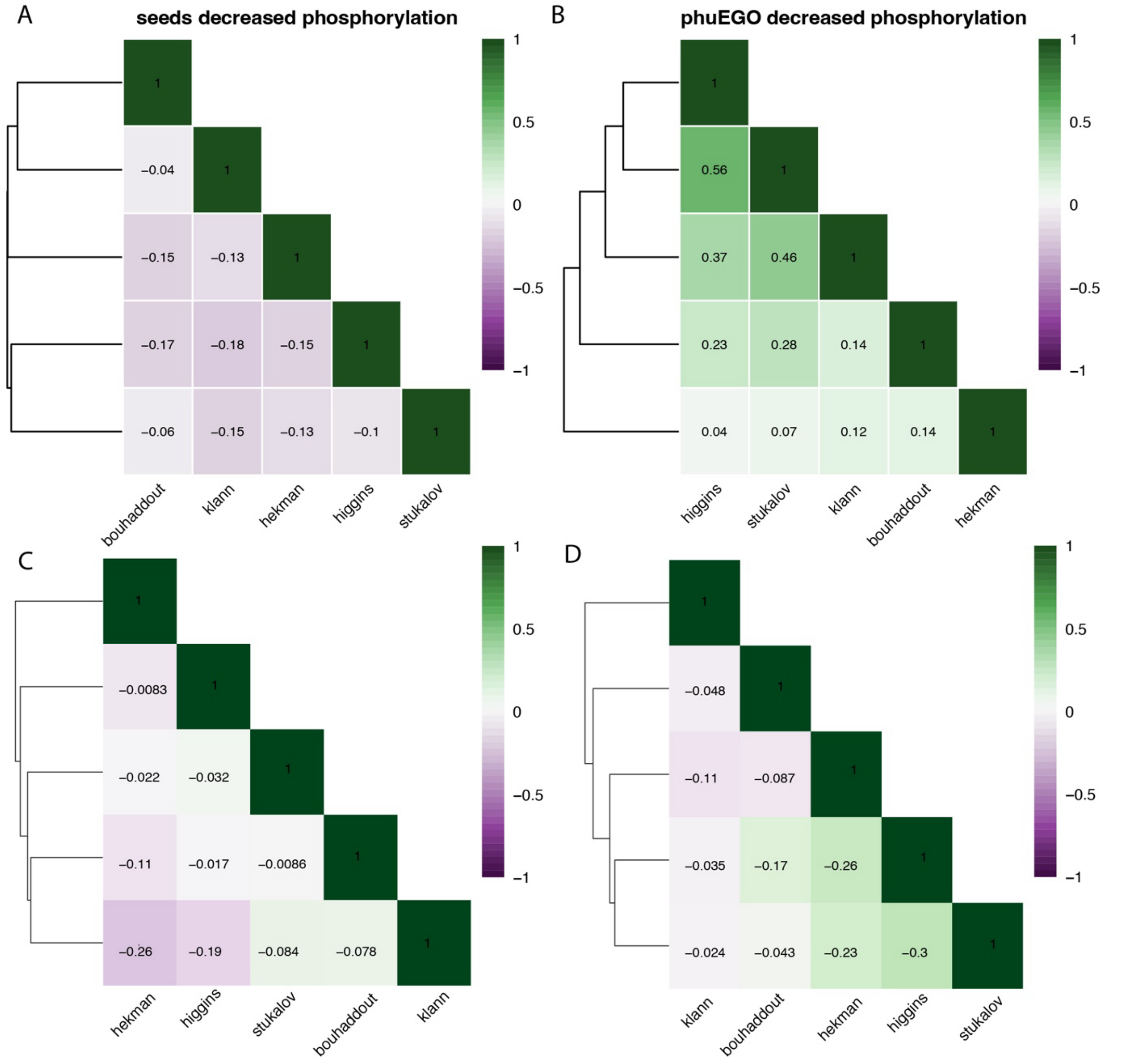

Supplementary Figure 9. Correlation of downregulated phosphosites in SARS-CoV-2 datasets A. before and **B.** after phuEGO. **C.** Correlation of upregulated kinases as estimated using KSEA **D.** Correlation of downregulated kinases as estimated using KSEA
